# *β-*catenin knockdown promotes NHERF1-mediated survival of colorectal cancer cells: implications for a double-targeted therapy

**DOI:** 10.1038/s41388-018-0170-y

**Published:** 2018-03-19

**Authors:** Concetta Saponaro, Sara Sergio, Antonio Coluccia, Maria De Luca, Giuseppe La Regina, Luca Mologni, Valeria Famiglini, Valentina Naccarato, Daniela Bonetti, Candice Gautier, Stefano Gianni, Daniele Vergara, Michel Salzet, Isabelle Fournier, Cecilia Bucci, Romano Silvestri, Carlo Gambacorti Passerini, Michele Maffia, Addolorata Maria Luce Coluccia

**Affiliations:** 1 Laboratory of Clinical Proteomics, Giovanni Paolo II Oncology Hospital, I-73100 Lecce, Italy; 2 Functional Biomorphology Laboratory, IRCCS Istituto Tumori “Giovanni Paolo II”, Bari, Italy; 3 Department of Biological and Environmental Sciences and Technologies, University of Salento, I-73100 Lecce, Italy; 4 Department of Drug Chemistry and Technologies, Sapienza University of Rome, Laboratory affiliated to Istituto Pasteur Italia – Fondazione Cenci Bolognetti, Piazzale Aldo Moro 5, I-00185 Roma, Italy; 5 Department of Clinical Medicine, San Gerardo Hospital, University of Milano-Bicocca, I-20052 Monza, Italy; 6 Department of Biochemistry, Sapienza University of Rome, Laboratory affiliated to Istituto Pasteur Italia – Fondazione Cenci Bolognetti, Piazzale Aldo Moro 5, I-00185 Roma, Italy; 7 U1192-Laboratoire Protéomique, Réponse Inflammatoire et Spectrométrie de Masse (PRISM), F-59000 Lille, France

## Abstract

Nuclear activated *β*-catenin plays a causative role in colorectal cancers (CRC) but remains an elusive therapeutic target. Using human CRC cells harboring different Wnt/*β*-catenin pathway mutations in *APC/KRAS* or *β-catenin/KRAS* genes, and both genetic and pharmacological knockdown approaches, we show that oncogenic *β*-catenin signaling negatively regulates the expression of NHERF1 (Na^+^/H^+^ exchanger 3 regulating factor 1), a PDZ-adaptor protein that is usually lost or downregulated in early dysplastic adenomas to exacerbate nuclear *β*-catenin activity. Chromatin immunoprecipitation (ChIP) assays demonstrated that *β*-catenin represses NHERF1 via TCF4 directly, while the association between TCF1 and the *Nherf1* promoter increased upon *β*-catenin knockdown. To note, the occurrence of a cytostatic survival response in settings of single *β*-catenin-depleted CRC cells was abrogated by combining NHERF1 inhibition via small hairpin RNA (shRNA) or RS5517, a novel PDZ1-domain ligand of NHERF1 that prevented its ectopic nuclear entry. Mechanistically, dual NHERF1/*β*-catenin targeting promoted an autophagy-to-apoptosis switch consistent with the activation of Caspase-3, the cleavage of PARP and reduced levels of phospho-ERK1/2, Beclin-1, and Rab7 autophagic proteins compared with *β*-catenin knockdown alone. Collectively, our data unveil novel *β*-catenin/TCF-dependent mechanisms of CRC carcinogenesis, also offering preclinical proof of concept for combining *β*-catenin and NHERF1 pharmacological inhibitors as a mechanism-based strategy to augment apoptotic death of CRC cells refractory to current Wnt/*β*-catenin-targeted therapeutics.

## Introduction

Nuclear activated *β*-catenin caused by Wnt-pathway mutations is the earliest driving event in hereditary (10%) and sporadic (90%) human colorectal cancers (CRC) [1]. Deletion of the tumor suppressor adenomatous polyposis coli (*APC*) (>80%) or ‘gain-of-function’ mutations in GSK3*β*-target residues in *β-catenin* gene (5–10%) increase the nuclear import of *β*-catenin with ensuing aberrant activation of TCF/LEF (T-cell factor/lymphoid enhanced factor) transcriptional factors. Activating mutations of *KRAS* and *BRAF* genes are detected in up to 60% of CRC, not long after *APC* or *β-catenin* mutations, and contribute to enforce the oncogenic signaling of *β*-catenin also conveying an invasive and metastatic behavior in cancer cells [2].

Despite its causative role in CRC, however, *β*-catenin remains an elusive therapeutic target. Targeting of *β*-catenin by small hairpin RNAs (shRNAs) or pharmacological inhibitors reduces the growth of established CRC xenografts in vivo without affecting the viability of malignant cells [3, 4]. CRC cells harboring inducible *β-catenin* shRNAs undergo cell cycle arrest, differentiate into polarized epithelial cells but rapidly resume their proliferative potential when relieved from *β*-catenin inhibition in vitro [5, 6]. Thus, from a biological and therapeutic perspective, a better characterization of the signaling outcome of *β*-catenin knockdown could provide new molecular events tied to survival control in CRC tumorigenesis that could be potentially used in combination therapy to improve the efficacy of Wnt-targeted approaches at early disease stages.

NHERF1 (Na^+^/H^+^ exchanger 3 regulating factor 1) is a membrane adaptor protein that contains two tandem PDZ (post-synaptic density 95/discs large/zona occludens 1) domains and physiologically recruits *β*-catenin underneath the plasma membrane to shape proper tissue morphogenesis and homeostasis [7]. Recent data earmark NHERF1 as a tumor suppressor upstream of Wnt/*β*-catenin-driven intestinal tumorigenesis in vivo [8]. The complete lack of NHERF1 expression, either by shRNA knockdown in CRC cells or by gene knockout (*NHERF1*^*−/−*^) in *Apc*^*Min/+*^ mutant mice that develop multiple intestinal adenomas, was reported to increase tumor growth and the transactivating effects of *β*-catenin [8]. These data have relatively explained the heterogeneous pattern of NHERF1 observed in primary CRC [9–11], where membranous expression of NHERF1 is usually lost in dysplastic adenomas, and either absent or low/ectopically re-expressed thorough the cytoplasm and nuclei in approximately half of differentiated adenocarcinomas [12, 13].

Little is known about molecular mechanisms governing the early loss-of-NHERF1 expression in CRC and, in particular, the impact of oncogenic *β*-catenin remains unexploited. Using CRC cells harboring different Wnt/*β*-catenin pathway mutations and low-NHERF1-levels at baseline, we show that genetic or pharmacological knockdown of *β*-catenin is sufficient to increase NHERF1 as a major driver of a cytoprotective autophagic response. This prompted us to investigate a double *β*-catenin/NHERF1-inhibitory strategy as a fruitful approach to augment apoptotic death of CRC cells refractory to Wnt-targeted agents, indicating RS5517 as a novel NHERF1/PDZ1-domain ligand antagonist with a promising therapeutic value.

## Results

### *β*-catenin represses NHERF1 expression by associating with TCF4

Mechanisms underlying the early lack of NHERF1 in CRC tumorigenesis remain unclear [7]. To determine the impact of *β*-catenin on NHERF1 expression, we used an integrated doxycycline (Dox)-inducible shRNA vector to silence *β*-catenin in two human cell lines that recapitulate the earliest events implicated in CRC (Ls174T have mutated *β-catenin/KRAS* genes and DLD-1 have mutated *APC/KRAS* genes) [14–16].

**Fig. 1:**
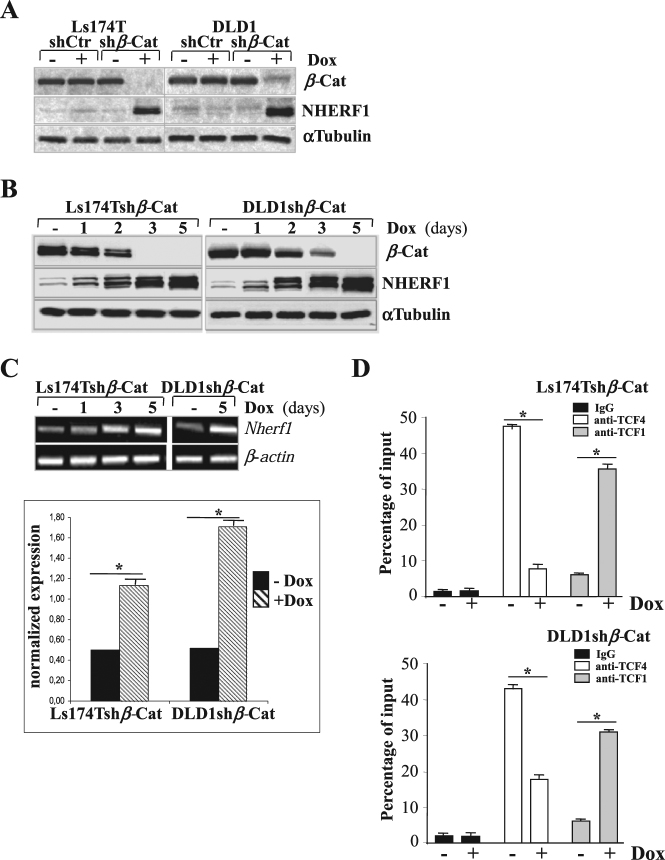
*β*-catenin represses NHERF1 expression through associating with TCF4. **a** Ls174T and DLD1 cells stably transfected with a doxycycline (Dox)-inducible small hairpin (sh)RNA for *β*-catenin (sh*β*-Cat) or a scramble shRNA (shCtr) as control were cultured without (−) or with (+) Dox for 5 days. Total cell lysates were then probed for *β*-catenin (*β*-Cat) and NHERF1 protein amounts. Tubulin served as a loading control. **b** Ls174Tsh*β*-Cat and DLD1sh*β*-Cat cells expressing a Dox-inducible shRNA for *β*-catenin were cultured without (−) or with (+) Dox for 1, 2, 3, or 5 days and then assessed by western blotting as indicated. **c** An equal amount of RNA (1 μg) of Ls174Tsh*β*-Cat and DLD1sh*β*-Cat cells cultured in the absence (−) or presence of Dox at the indicated time points, was analyzed by RT-PCR for assessing *nherf1* and β-actin mRNA levels. The normalized *nherf1*expression versus β-actin mRNA at day 5 was quantified by using Image J analysis software for Windows. Data from three independent experiments were presented as means ± SEM (*p < 0.05). **d** ChIP of TCF1 and TCF4 in the *Nherf1* promoter region in Ls174Tsh*β*-Cat and DLD1sh*β*-Cat cells cultured in the absence (−) or presence of Dox (+) for 5 days. qPCR was performed to quantify ChIP assay results. Enrichment was quantified relative to input controls. An anti-IgG antibody was used as a negative control. Results are represented as the average ± SD of three independent experiments (*p < 0.01)

*β*-catenin was successfully silenced in sh*β*-catenin stably transfected Ls174T and DLD-1 cells (below named Ls174Tsh*β*-Cat and DLD1sh*β*-Cat) cultured with Dox (+Dox) compared with untreated cells (−Dox) or a mock clone expressing a negative control shRNA (shCtr) (Fig. 1a, b). Both CRC cell lines expressed low-steady-state levels of NHERF1 (Fig. 1b, c), while its mRNA transcript and protein levels was gradually increased in sh*β*-Cat-depleted CRC cells maintained under Dox (+Dox), achieving a peak of induction upon complete *β*-catenin knockdown (Fig. 1b, c). Then we performed ChIP assays to determine whether *β*-catenin regulates NHERF1 via recruiting key transcriptional mediators of the Wnt pathway like TCF1, also called transcription factor 7 (TCF7), and TCF4 also called transcription factor 7-like 2 (TCF7L2), both of which involved in CRC carcinogenesis [1, 2]. We observed that knockdown of *β*-catenin strikingly reduced the binding of TCF4 with the *Nherf1* promoter, while promoting the association with TCF1 in both CRC cell lines (Fig. 1d)​.

**Fig. 2:**
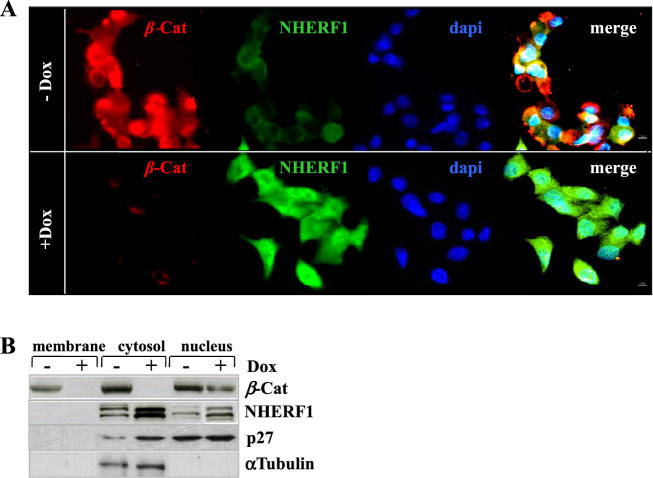
Ectopic cytoplasmic and nuclear overexpression of NHERF1 in *β*-catenin-depleted CRC adenocarcinoma cells. **a** Ls174Tsh*β*-Cat were cultured in the absence or presence of Dox (−Dox/+Dox) for 5 days, stained with primary antibodies for *β*-catenin (*β*-Cat) or NHERF1 and visualized with a fluorescent IgG-TRITC (for *β*-Cat) or IgG-FITC (for NHERF1) secondary antibodies by laser scanning confocal microscopy. DNA was stained with 4′,6-diamidino-2-phenylindole (DAPI). A merge of the two fluorescence signals and DAPI staining is also shown (Magnification, ×60). Each confocal image is representative of three independent experiments. **b** DLD1sh*β*-Cat cells were cultured in the absence or presence of 2 μg/mL of Dox (−Dox/+Dox) for 5 days and then fractionated to obtain purified extracts from either membrane, cytosol, and nucleus of cells, as described in Methods section. Equal amounts of each cell extract (100 μg) were separated by SDS-PAGE and probed with the indicated antibodies

We also examined the intracellular distribution of NHERF1 and *β*-catenin in Ls174Tsh*β*-Cat cells by laser scanning confocal microscopy (Fig. 2a). β-catenin was nearly undetectable in Dox-treated cells correlating with a more pronounced ectopic staining of NHERF1 compared with the Dox-untreated control samples (Fig. 2a). Similar results were obtained by testing DLD1sh*β*-Cat cells (data not shown), which were also subjected to cell fractionation experiments to assess changes on NHERF1 subcellular compartmentalization during *β*-catenin knockdown (Fig. 2b). A fraction of cortical *β*-catenin was evident in Dox-untreated CRC cells (−Dox), although it was predominantly accumulated in the cytosolic and nuclear fractions, in line with an oncogenic *β*-catenin/TCF-transcriptional activation. Following Dox-exposure (+Dox), *β*-catenin remained evident only in the nuclear extracts with ensuing increased cytoplasmic/nuclear NHERF1 levels vs Dox-untreated (−Dox) cells.

**Fig. 3:**
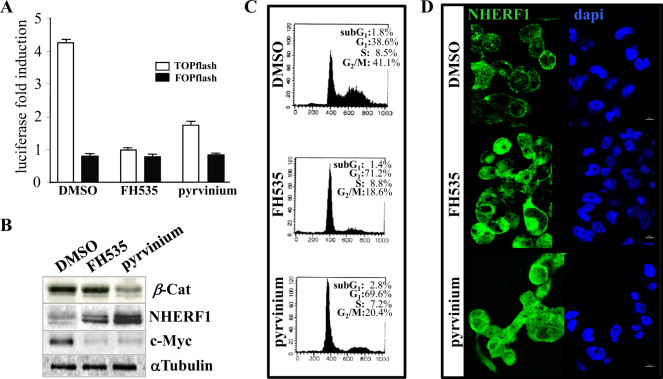
Small molecule inhibitors of *β*-catenin promote NHERF1 overexpression and mislocalization in CRC cells. **a** Subconfluent Ls174Tsh*β*-Cat were co-transfected with the luciferase reporter constructs TOPflash or FOPflash as reported in Methods section. At 24 h after transfection, cells were treated with vehicle alone (DMSO), FH535 (1 μM) or pyrvinium pamoate (150 nM) for additional 72 h. Luciferase activity was expressed as fold activation compared with carrier control cells. Values represent means ± SD of three representative experiments. **b** After 4 days of treatment with DMSO, FH535 (1 μM) or pyrvinium pamoate (150 nM), total cell lysates were probed for *β*-catenin (*β*-Cat), c-Myc and NHERF1 expression. Tubulin served as a loading control. **c** Ls174Tsh*β*-Cat cells exposed for 4 days to DMSO, FH535 (1 μM) or pyrvinium pamoate (150 nM) were analyzed by propidium iodide-staining and flow cytometric analysis for assaying cell cycle distribution, as described in Methods section. A representative dot plot of three replicate experiments shows the percentages of cells into different phases of the cell cycle for each indicated treatment. **d** Each confocal image is representative of three indipendent immunofluorescence analyses of NHERF1 by a rabbit polyclonal antibody visualized with a fluorescent IgG-FITC secondary antibody for the indicated treatments (left panels). DAPI staining (right panels) is also shown (Magnification, ×60)

We also observed that NHERF1 was significantly upregulated in Ls174Tsh*β*-Cat cells treated with FH535 and pyrvinium pamoate, two unrelated drugs that have been reported to block *β*-catenin signaling via different mechanisms (Fig. 3). FH535 is a sulfonamide-based compound that suppresses *β*-catenin/TCF-mediated transcription without affecting *β*-catenin levels [17]. Pyrvinium is an anthelmintic drug that promotes *β*-catenin degradation via activation of CK1α (casein kinase1α) [18]. Both drugs reduced transcription from the *β*-catenin/TCF-responsive reporter plasmid TOPflash (Fig. 3a) and expression of c-Myc, a *β*-catenin transcriptional target that promotes cell proliferation (Fig. 3b). The two drugs induced CRC cell cycle arrest without significant apoptosis (Fig. 3c) [5, 6] and increased low-endogenous NHERF1 levels, as well as its ectopic cytoplasmic/nuclear subcellular distribution, although at a higher extent in pyrvinium vs FH535-treated cells (Fig. 3b, d).

To our knowledge, these data unveils a new oncogenic role for *β*-catenin in CRC through very early NHERF1 suppression at the transcriptional level via TCF4 directly.

### Genetic targeting of *β*-catenin and NHERF1 causes massive CRC apoptosis

**Fig. 4:**
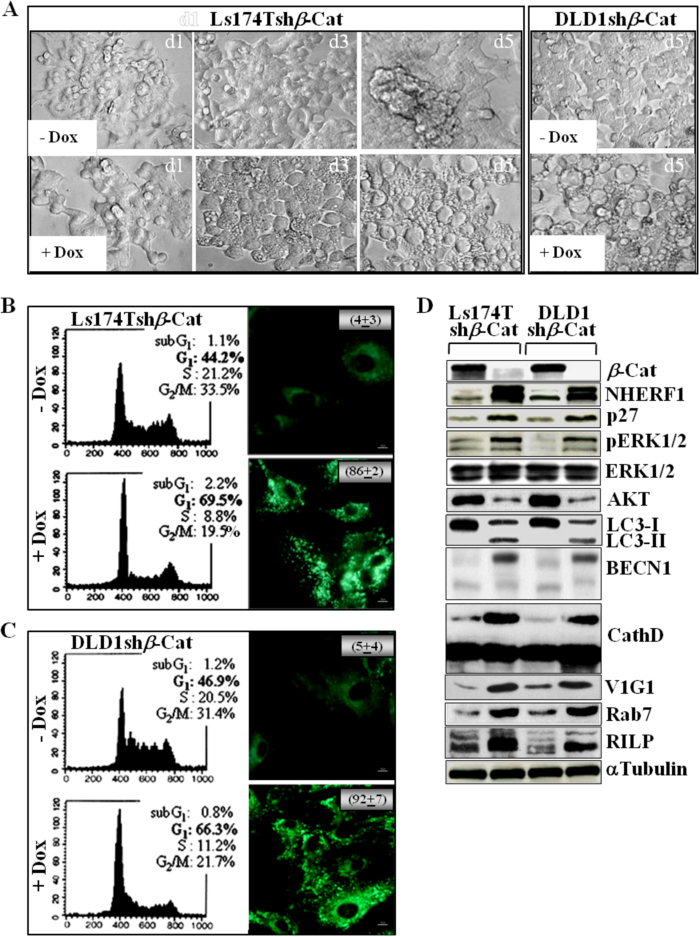
*β*-catenin-silenced CRC cells show a unique protein profile centred around autophagy and energy metabolism. Ls174T and DLD1 cells expressing a doxycycline (Dox)-inducible shRNA for *β*-catenin (Ls174Tsh*β*-Cat and DLD1sh*β*-Cat) were cultured without or with 2 μg/mL of Dox (−Dox/+Dox) for 1, 3, or 5 days. **a** A representative image of cells from one of the six fields captured from each well by using a camera attached to an inverted Olympus IX51 microscope is shown. **b** After 5 days of treatment without or with doxycycline (−Dox/+Dox), Ls174Tsh*β*-Cat cells were analyzed by propidium iodide-staining and flow cytometric analysis for assaying cell cycle distribution, as described in Methods section. A representative dot plot of three replicate experiments shows the percentages of cells into different phases of the cell cycle for each indicated treatment (left panels). Under the same experimental conditions, Ls174Tsh*β*-Cat cells were analyzed for the presence of MDC-positive autophagosomes, distinct dot-like structures trapped in acidic, membrane-rich organelles distributed in the cytoplasm or localizing in the perinuclear regions of CRC cells (Magnification, ×60). The numbers indicate the percentage of autophagy induction for each treatment condition. **c** After 5 days of treatment without or with doxycycline (−Dox/+Dox), cell cycle distribution of DLD1sh*β*-Cat cells was analyzed by propidium iodide-staining and flow cytometric analysis. A representative plot of three replicate experiments shows the percentages of cells into different phases of the cell cycle for each indicated treatment (left panels). DLD1sh*β*-Cat cells were analyzed for the presence of MDC-positive autophagosomes, as previously reported for Ls174Tsh*β*-Cat cells (Magnification, ×60). The numbers indicate the percentage of autophagy induction for each treatment condition. **d** Total cell lysates from Ls174Tsh*β*-Cat and DLD1sh*β*-Cat cultured without (−) or with (+) Dox for 5 days were assessed by western blotting with the indicated antibodies

Knockdown of *β*-catenin promoted cell differentiation coupled to a prominent vacuolization in Dox-treated (+Dox) Ls174Tsh*β*-Cat and DLD-1sh*β*-Cat cells vs their untreated counterparts (−Dox) (Fig. 4a) [5, 6]. These changes were reversible on Dox removal (data not shown). Similarly to pharmacological targeting of *β*-catenin (Fig. 3), genetic depletion of *β*-catenin was sufficient to cause cell cycle arrest (Fig. 4b, c, left panels) sustaining an adaptive autophagic response, as judged by staining for monodansylcadaverine (MDC) used as a hallmark of autophagic vacuoles (Fig. 4b, c, right panels). As shown in Fig. 4d, heightened endogenous levels of NHERF1 in settings of viable *β*-catenin-depleted CRC cells also correlated with hyper-phosphorylated ERK1/2 (pERK1/2) and increased levels of the cell cycle inhibitor p27, as well as of key endocytosis/autophagy regulators, such as the microtubule-associated protein light chain 3 (LC3), Beclin-1 (BECN1), Cathepsin D, the regulatory V1G1 subunit of the vacuolar ATPase (V-ATPase), the small GTPase Rab7 and its effector RILP [19, 20]. Specifically, the accumulation of LC3-II, Beclin-1, and Cathepsin D isoforms were specific molecular makers of autophagy predicted based on morphological parameters (Fig. 4a). These findings were corroborated by an unbiased broad proteomic characterization indicating that 1161 proteins were differentially expressed in Ls174Tsh*β*-Cat cells cultured with or without doxycycline (±Dox) (Supplementary Figure S1). The heat-map generated by Perseus segregated samples into two separated branches characterized respectively by 464 proteins upregulated (Cluster 1) and 697 proteins downregulated (Cluster 2) in *β*-catenin-depleted cells. Each protein cluster was also enriched with specific signaling cascades based on KEGG (Kyoto Encyclopedia of Genes and Genomes) pathway analysis using STRING software (Table 1). These proteins were statistically categorized by STRING (false discovery rate ≤1.31e−05) showing that Cluster 1 was enriched with proteins involved in the assembling of actin cytoskeleton and adherens junctions, fatty acid degradation, phagosome/lysosome maturation, autophagy, and endocytosis. Conversely, downregulated proteins into the Cluster 2 were involved in the regulation of spliceosome, RNA transport and ribosome biogenesis. This proteomic profiling corroborated the molecular changes reported in Fig. 4d, earmarking differences into our dataset in terms of energy metabolism, actin filament-based processes and especially autophagic/endocytic processes.

**Table 1:** The principle KEGG pathways of the upregulated and downregulated genes

	Category	Pathway ID	Pathway description	Count in gene set	False discovery rate
Upregulated	KEGG_ PATHWAY	01100	Metabolic pathways	80	4.79e−17
	KEGG_ PATHWAY	04810	Regulation of actin cytoskeleton	24	8.5e−09
	KEGG_ PATHWAY	05132	Salmonella infection	15	3.03e−08
	KEGG_ PATHWAY	04145	Phagosome	19	3.45e−08
	KEGG_ PATHWAY	04721	Synaptic vesicle cycle	13	3.6e−08
	KEGG_ PATHWAY	05110	Vibrio cholerae infection	12	4.97e−08
	KEGG_ PATHWAY	04142	Lysosome	17	6.65e−08
	KEGG_ PATHWAY	04144	Endocytosis	20	5.05e−07
	KEGG_ PATHWAY	05130	Pathogenic *Escherichia coli* infection	11	5.54e−07
	KEGG_ PATHWAY	00071	Fatty acid degradation	10	7.72e−07
	KEGG_ PATHWAY	04530	Tight junction	17	7.72e−07
Downregulated	KEGG_ PATHWAY	03040	Spliceosome	50	1.71e−38
	KEGG_ PATHWAY	03013	RNA transport	40	1.06e−22
	KEGG_ PATHWAY	03008	Ribosome biogenesis in eukaryotes	27	1.68e−19
	KEGG_ PATHWAY	03010	Ribosome	34	1.68e−19
	KEGG_ PATHWAY	01100	Metabolic pathways	96	1.2e−14

A high confidence (0.700) was set as the threshold to define significant differences

**Fig. 5:**
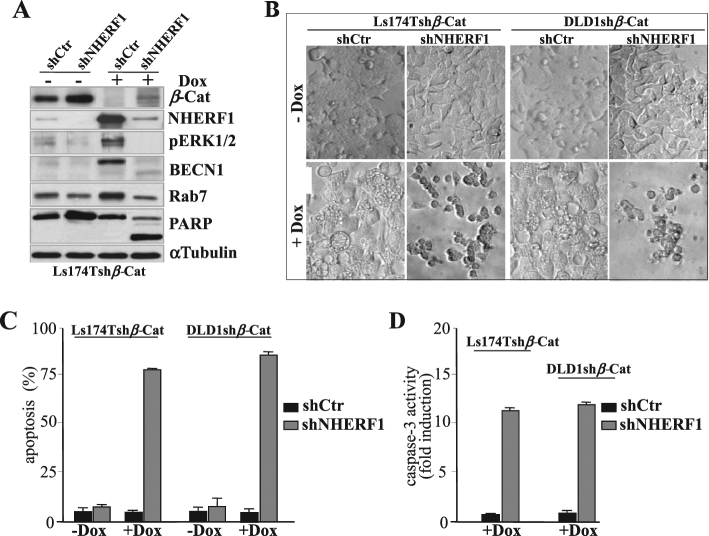
Combined shRNA-mediated downregulation of *β*-catenin and NHERF1-induces apoptosis in human CRC cells. Subconfluent Ls174T cells stably harboring a doxycycline (Dox)-inducible shRNA for *β*-catenin (Ls174Tsh*β*-Cat) were further transiently transfected with 200 nM of NHERF1 targeted shRNAs (shNHERF1) or scramble shRNAs as control (shCTR). **a** Total protein extracts from double sh*β*-Cat/shCTR or sh*β*-Cat/shNHERF1 expressing cells cultured without (−) or with (+) Dox for 5 days to simultaneously induce shRNAs for *β*-catenin were assessed by western blotting with the indicated antibodies. **b** A representative image of Dox-treated (+Dox) or untreated shCTR or shNHERF1 expressing Ls174Tsh*β*-Cat or DLD1sh*β*-Cat cells from one of the six fields captured from each well by using a camera attached to an inverted Olympus IX51 microscope is shown. **c** Ls174Tsh*β*-Cat or DLD1sh*β*-Cat cells expressing a scramble control shCTR or specific shRNAs for NHERF1 (shNHERF1) were cultured without (−) or with (+) Dox for 5 days and then labeled by Annexin V/PI (propidium iodide) staining for apoptosis evaluation by flow cytometry. Values represent the mean ± SD of three independent experiments. **d** Caspase-3 activity was also measured and data reported as fold induction over vehicle-treated control samples

Overall these considerations prompted us to investigate whether NHERF1 could play a role in modulating ERK1/2 and Rab7 expression upon *β*-catenin depletion (Fig. 5). To this end, Ls174Tsh*β*-Cat or DLD1sh*β*-Cat cells were transiently transfected with NHERF1 targeted shRNAs (shNHERF1) or scramble shRNAs as control (shCtr) and then cultured with or without Dox (±Dox) to modulate concomitantly stable si*β*-catenin expression. As shown in Fig. 5a, NHERF1 knockdown alone did not alter overall phospho-ERK1/2, Beclin-1 (BECN1), Rab7, and PARP protein levels at baseline, while combined targeting of NHERF1 and *β*-catenin annulled the hyper-activation of ERK1/2 and the accumulation of the autophagy markers Beclin-1 (BECN1) and Rab7 elicited by single*-β*-catenin depletion (Fig. 5a, 4d). Such molecular changes were consistent with a massive apoptotic death occurrence in Dox-treated Ls174Tsh*β*-Cat/shNHERF1 (75 ± 6%) and DLD1sh*β*-Cat/shNHERF1 (82 ± 8%) cells, compared with <5% in controls (sh*β*-Cat/siCtr+Dox or sh*β*-Cat/shNHERF1−Dox) (Fig. 5b, c), as further confirmed by the cleavage of PARP (Fig. 5a) and Caspase-3 activity (Fig. 5d). Interestingly, we also noticed a modest increase of *β*-catenin protein amounts in sh*β*-Cat/shNHERF1 maintained with or without Dox for 4 days (Fig. 5a), in keeping with additive affects that NHERF1 knockdown extents on *β*-catenin stabilization reported by others [8]. Moreover, NHERF1 knockdown alone conferred a moderate clonogenic advantage to Dox-untreated Ls174Tsh*β*-Cat or DLD1sh*β*-Cat cells (Supplementary Figures S2A and S2B) which showed a more sprouted/invasive phenotype when cultured in sub-confluent monolayers (Fig. 5b) or in soft agar (Supplementary Figure S2C).

Collectively, these data indicate that combining NHERF1 inhibition in settings of *β*-catenin-silenced CRC cells, in which NHERF1 is highly/ectopically re-expressed, is mandatory to promote an autophagy-to-apoptosis switch in CRC cells refractory to single*-β*-catenin targeting.

### A novel NHERF1/PDZ1-domain antagonist prevents its nuclear import and synergizes with *β*-catenin inhibitors in promoting CRC apoptosis

**Fig. 6:**
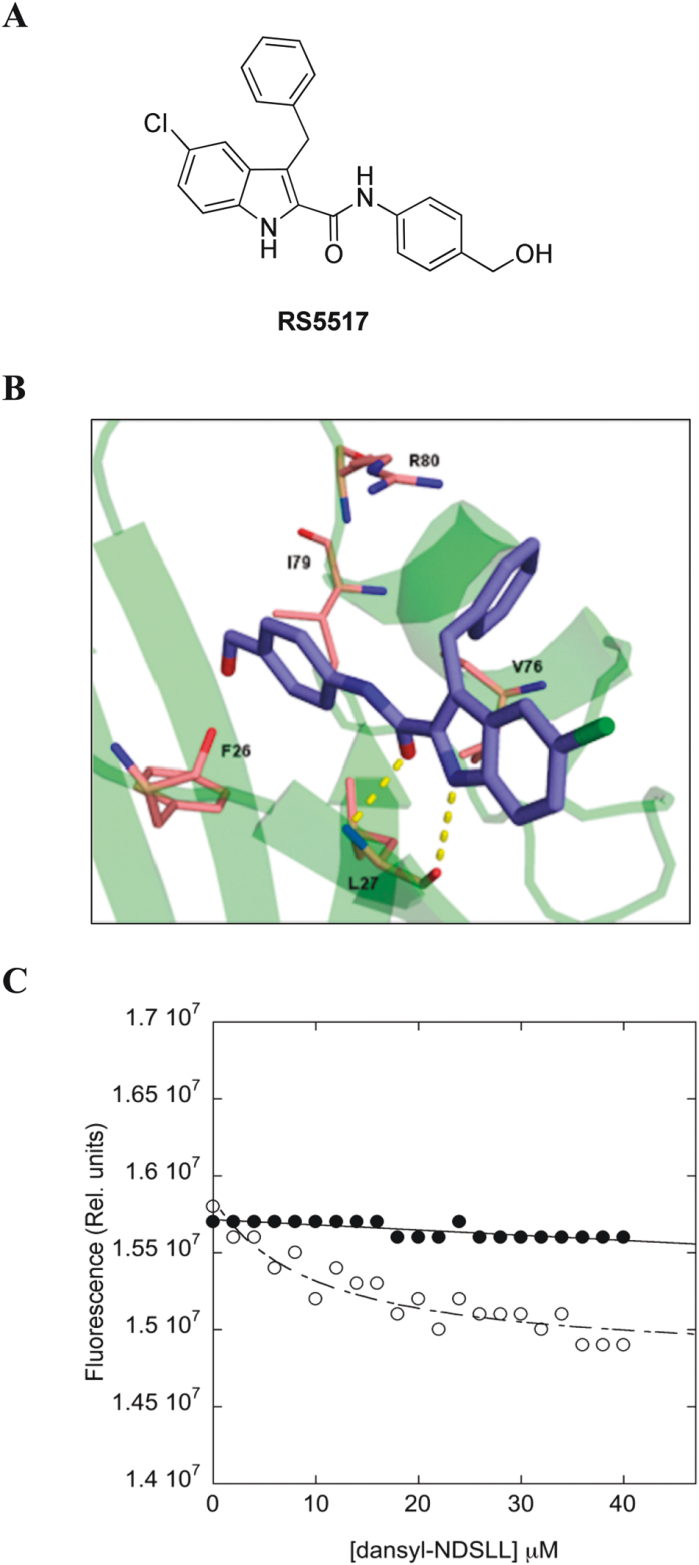
RS5517 is a new PDZ1-domain antagonist of NHERF1. **a** The chemical structure of RS5517 derivative. **b** Proposed binding mode for derivative RS5517 (purple) to the NHERF1 PDZ1 domain that is depicted as green cartoon. Residues involved in interactions were reported as pink stick. H-bonds were shown as yellow dot lines. **c** Binding of PDZ1 NHERF1 Y38W to the ligand Dansyl-NDSLL in the presence (filled circles) and in the absence (open circles) of 5 mM RS5517. Fluorescence data were recorded in the presence of 50 mM Na phosphate, pH 7.2, 300 mM NaCl, 5 mM DTT, 20% DMSO, at 25 °C. Lines are the best fir to a hyperbolic binding transition. It is evident that binding between PDZ1 NHERF1 Y38W to the ligand Dansyl-NDSLL is abolished in the presence of RS5517

Previous studies focused on small molecule ligands of the PDZ1-domain of NHERF1 to prevent its ectopic cytoplasmic/nuclear mislocalization and oncogenic function [21–23]. These studies predicted the canonical inactivating binding motif for the NHERF1 PDZ1-domain on four D/E-(S/T)-X-(L/V/I/M) residues that are commonly contained at the C-terminal of its specific ligands, such as DSLL for the β2-adrenergic receptor (β2-AR) or ETWM for the parathyroid hormone receptor (PTHR) [24]. Based on these premises, we herein evaluated the conformational flexibility of NHERF1 PDZ1-DSLL and PDZ1-ETWM peptide complexes by molecular dynamics (MD) simulations to draw a consensus pharmacophore model, as described in Materials and Methods [25, 26]. An in-house library of 6000 compounds was screened using the pharmacophore model leading to the selection of RS5517 as the only one of six lowest-energy derivatives with remarkable cytotoxicity in vitro (Fig. 6 and data not shown).

The chemical structure of RS5517, namely 3-benzyl-5-chloro-N-(4-(hydroxymethyl)phenyl)-1H-indole-2-carboxamide, is depicted in Fig. 6a. The analysis of the binding mode of RS5517 led us to identify a series of residues involved in the interactions at the NHERF1 PDZ1 domain (Fig. 6b). Specifically: (i) a key Pi-cation interaction between the R80 guanidine and the benzyl group at position 3 of the indole of RS5517; (ii) hydrophobic contacts between the 2-(4-hydroxymethyl)benzoyl moiety and F27 and I79 of the PDZ1, and between the indole nucleus and V76; (iii) two H-bonds with L27 backbone.

To address the inhibitory effect of RS5517, we resorted to test the effect of this molecule on NHERF1/PDZ1 domain in vitro. A construct encoding for NHERF1 PDZ1 was expressed and challenged with a dansylated peptide corresponding to the C-terminal sequence of β2-AR (D-NDSLL). In order to measure binding, a fluorescent pseudo-wild type was produced by replacing Tyr38 of PDZ1 with a Trp. Binding was then measured by Forster Resonance energy transfer between the fluorescent donor (Trp) and acceptor (Dansyl). Binding experiments were performed in the presence and in the absence of a constant concentration of RS5517 (Fig. 6c). It is evident, that, while the PDZ1 is capable to bind the C-terminal sequence of β2-AR with an affinity of about 10 μM, binding is essentially abolished in the presence of RS5517. This finding confirms that RS5517 is a specific inhibitor of NHERF1/PDZ1 domain.

**Fig. 7:**
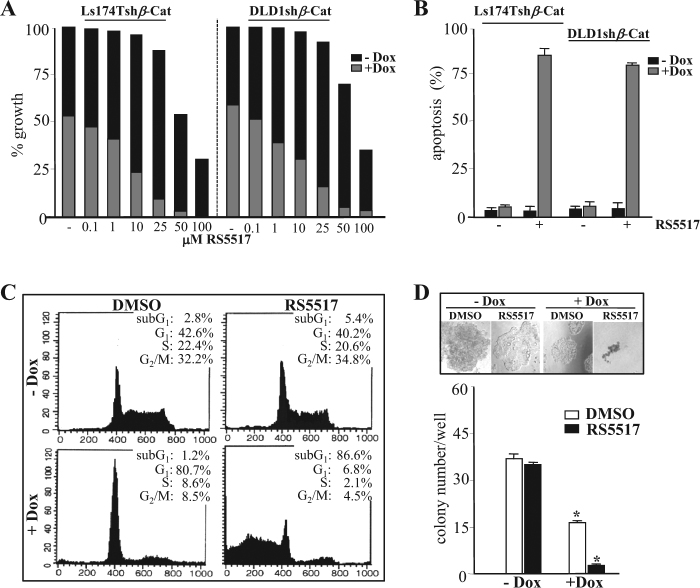
RS5517 synergizes with shRNA-mediated silencing of *β*-catenin in promoting CRC cell death. **a** Proliferation assays of Ls174Tsh*β*-Cat or DLD1sh*β*-Cat cells exposed to increasing concentrations of RS5517 (range 0.1–100 μM) or vehicle alone (−) in the absence or concomitant presence of Dox (−Dox/+Dox) for 5 days. Data were expressed as % of growth inhibition. **b** Ls174Tsh*β*-Cat or DLD1sh*β*-Cat cells were treated with 10 μM RS5517 in the absence or presence of Dox (−Dox/+Dox) for 5 days and then labelled by Annexin V-PE for apoptosis evaluation by flow cytometry. Values represent the mean ± SD of three independent experiments. **c** Ls174Tsh*β*-Cat or DLD1sh*β*-Cat cells were treated with 10 μM RS5517 (+) or DMSO as vehicle control (−) in the absence or concomitant presence of Dox (−Dox/+Dox) for 5 days and then analyzed by propidium iodide-staining and flow cytometric analysis for assaying cell cycle distribution, as described in Methods section. A representative dot plot of three replicate experiments shows the percentages of cells into different phases of the cell cycle for each indicated treatment. **d** Number of Ls174Tsh*β*-Cat or DLD1sh*β*-Cat colonies formed in soft agar containing DMSO or 10 μM RS5517 after 14 days. The values are presented as mean ± SD of three independent experiments (*P < 0.05). A representative cell colony image was captured for the indicated treatment condition by using a camera attached to an inverted Olympus IX51 microscope

To evaluate the growth inhibitory effects of RS5517, Ls174Tsh*β*-Cat, or DLD1sh*β*-Cat cells were exposed to escalating doses (0.1–100 μM) of the compound in the presence or absence of Dox for 4 days and then analyzed by MTT assays. As shown in Fig. 7a, the half maximal inhibitory concentration (IC_50_) for RS5517 in Dox-untreated Ls174Tsh*β*-Cat or DLD1sh*β*-Cat cells expressing low levels of NHERF1 was 52 ± 6 μM and 65 ± 11 μM, respectively. Conversely, the equivalent IC_50_ value of RS5517 was reduced to 8 ± 2 μM in Ls174Tsh*β*-Cat cells and 11 ± 5 μM in DLD1sh*β*-Cat cells maintained under concomitant Dox exposure. Our mechanistic investigations suggest that the enhanced cytotoxic effects of RS5517 against Dox-treated CRC cells can be explained by inhibition of a NHERF1-mediated survival response restricted to cells that silence *β*-catenin (Fig. 5). Moreover, RS5517 used as single agent did not substantially affect the survival, proliferation rate and clonogenic potential of sh*β*-Cat transfected CRC cells (Fig. 7b, c). On the contrary, the combined use of 10 μM RS5517 and Dox increased the proportion of apoptotic cells in the sub-G_1_ peak of the cell cycle compared with single-Dox exposure (86.6 vs 5.4%) (Fig. 7c), by also impairing the clonogenicity of *β*-catenin-depleted cells (Fig. 7d). Interestingly, RS5517 alone did not confer any proliferative advantage to CRC cells compared with shRNA-based NHERF1 knockdown (Supplementary Figure S2), while promoting the outgrowth of more cohesive cell colonies in soft agar (Fig. 7d).

**Fig. 8:**
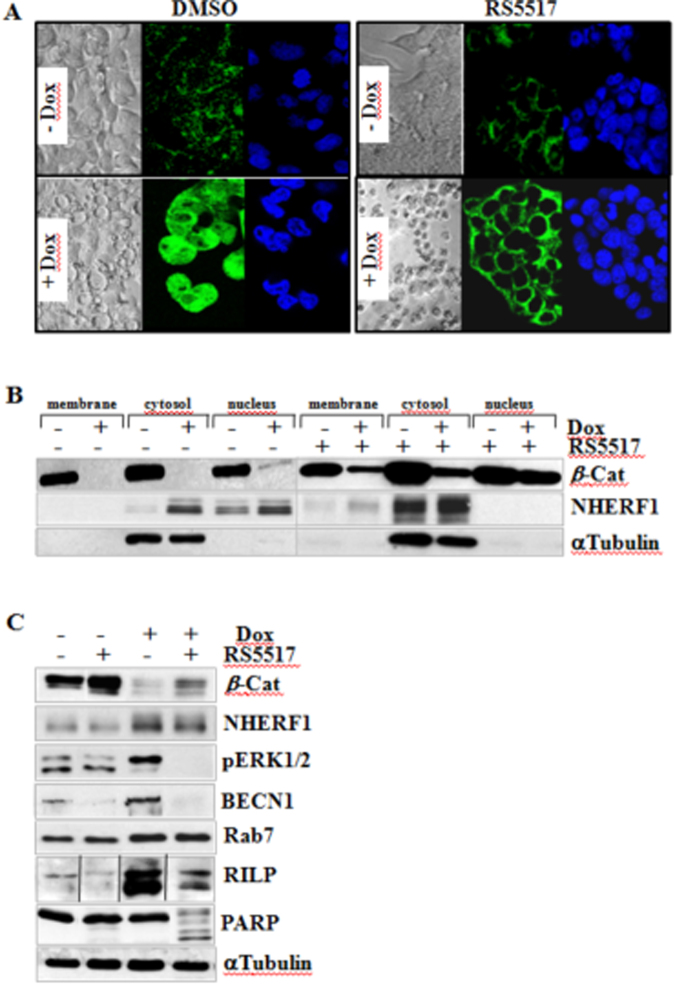
RS5517 prevents nuclear import of NHERF1 and sh*β*-catenin-mediated accumulation of phospho-ERK1/2 and RILP in CRC cells. **a** DLD1sh*β*-Cat cells treated with 10 μM RS5517 or DMSO in the absence or concomitant presence of Dox (−Dox/+Dox) for 5 days were stained using an anti-NHERF1 rabbit polyclonal primary antibody followed by an IgG-FITC secondary antibody and DAPI staining for each indicated treatment. Each confocal image is representative of three independent immunofluorescence experiments (Magnification, ×60). **b** Ls174Tsh*β*-Cat cells were treated with 10 μM RS5517 or DMSO in the absence or concomitant presence of 2 μg/mL of Dox (−Dox/+Dox) for 5 days and then fractionated to obtain purified extracts from either membrane, cytosol, and nucleus of cells, as described in Methods section. Equal amounts of each cell extract (100 μg) were separated by SDS-PAGE and probed with the indicated antibodies. **c** Ls174Tsh*β*-Cat cells were exposed to 10 μM RS5517 in the absence or concomitant presence of 2 μg/mL of Dox (−Dox/+Dox) for 5 days and total lysates were then analyzed by western blotting with the indicated antibodies

Confocal immunofluorescence and cell fractionation analysis revealed that RS5517 markedly prevented the nuclear import of NHERF1 in CRC cells cultured in the absence or presence of Dox restoring its physiological membranous localization (Fig. 8a, b). Moreover, RS5517 did not change NHERF1 levels whilst it had stabilizing effects on the overall *β*-catenin protein content regardless of Dox treatment (Fig. 8b, c). To further exploit this issue, DLD1sh*β*-Cat cells were prior exposed to RS5517 and/or Dox for 3 days, then labeled with [^35^S]-methionine and chased with complete non-radioactive medium for additional 4 or 8 h to analyze metabolic stability of *β*-catenin by pull-down assays (Supplementary Figure S3). RS5517 delayed the protein degradation rate of *β*-catenin, as judged by densitometric analysis (expressing the intensity of the labeled *β*-catenin bands as a percentage of the value at time 0) indicating that the estimated half-life of *β*-catenin was longer than 8 h in RS5517-treated samples over DMSO control samples (−). The occurrence of apoptotic cell death in Ls174sh*β*-Cat cells exposed to Dox and RS5517 (Fig. 7) was further confirmed by the cleavage of PARP and reduced phospho-ERK1/2, Beclin-1 (BECN1) and the Rab-effector RILP protein levels compared to cells exposed to single-Dox treatment (i.e., only *β*-catenin silenced). Intriguingly, the RS5517-mediated targeting of NHERF1 did not affect the overall Rab7 expression neither at baseline or during *β*-catenin knockdown (Dox setting), in contrast to what herein reported for shRNA-mediated NHERF1 knockdown (Figs. 8c and 5a).

**Fig. 9:**
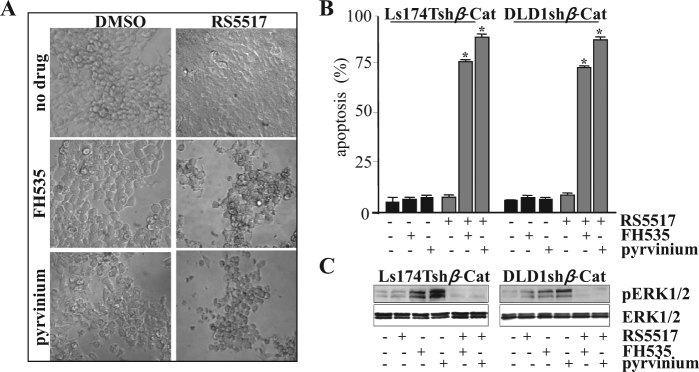
RS5517 induces CRC cell death when combined to pharmacological inhibitors of *β*-catenin. **a** Ls174Tsh*β*-Cat cells were treated with 10 μM RS5517 as single agent or in combination with FH535 (1 μM) or pyrvinium pamoate (150 nM) for 4 days and a representative image of one of the six fields captured from each well by using a camera attached to an inverted Olympus IX51 microscope is shown.** b** Ls174Tsh*β*-Cat or DLD1sh*β*-Cat cells treated with 10 μM RS5517 alone or in combination with FH535 (1 μM) or Pyrvinium pamoate (150 nM) for 4 days were then labelled by Annexin V/propidium iodide staining for apoptosis evaluation by flow cytometry. Values represent the mean ± SD of three independent experiments (*P < 0.003). **c** Total lysates from Ls174Tsh*β*-Cat or DLD1sh*β*-Cat cells treated with 10 μM RS5517 alone or in combination with FH535 (1 μM) or Pyrvinium pamoate (150 nM) for 4 days were analyzed by western blotting with the indicated antibodies

We finally tried to recapitulate our previous findings by the use of RS5517 and small-molecule inhibitors of *β*-catenin (Fig. 9). We show here that RS5517 synergized with *β*-catenin inhibitors FH535 and pyrvinium pamoate in triggering massive apoptosis of Ls174T and DLD1 colon cancer cells (Fig. 9a, b), thereby preventing persistent ERK1/2 phospho-activation mediated by single-*β*-catenin targeting (Fig. 9c).

## Discussion

The PDZ-scaffold protein NHERF1 is over-expressed in breast and colon carcinomas [12–14], associated significantly with aggressive histological grade and poor prognosis [27–31]. By physically tethering several cancer-related proteins, such as *β*-catenin [8], the tyrosine-kinase receptors PDGFR (platelet-derived growth factor receptor) [32] and EGFR (epidermal growth factor receptor) [33], some Wnt-ligand engaged receptors of the Frizzled (FZD) family [34], the tumor suppressor phosphatases PTEN (phosphatase and tensin homolog) [35] or PHLPP (PH leucine-rich repeat protein phosphatase) [36], NHERF1 engages in various functional complexes promoting specific phenotypic outcomes in cancer progression and metastatic organotropism [13, 27, 31]. However, NHERF1 is early lost and weakly re-expressed during the adenoma-to-adenocarcinoma transition [8, 12, 27, 35], thus playing potential antinomic roles as tumor suppressor, when it maintains a proper membrane localization, or tumor promoter when its expression is lost or mislocalized in the cytoplasm and nucleus [7, 9].

Loss of heterozygosity at the *NHERF1* gene locus (17q25.1) or somatic intragenic missense mutations occur in the majority of human ovarian and breast cancers but not other diseases examined to date [37]. The promoter of *NHERF1* gene contains estrogen-responsive elements [38], and NHERF1 expression was correlated with increasing ER (estrogen receptor) levels in >90% of ER-positive breast carcinomas, while it is absent in ER-negative tumors associated with early recurrence and poor survival [39].

Regarding CRC, a recent study stated the tumor suppressor activity of NHERF1 [7, 8]. NHERF1 depletion exacerbated the transformed phenotype in vitro and in vivo, thereby increasing nuclear *β*-catenin signaling [8]. The present study extends these findings showing that mRNA transcript and protein levels of NHERF1 expression are negatively regulated by oncogenic *β-*catenin signaling in CRC cell lines harboring different Wnt/*β*-catenin pathway mutations. Intriguingly, ChIP assays demonstrated that nuclear activated *β*-catenin can regulate NHERF1 via TCF4 directly, while the interaction between TCF1 and the *nherf1* promoter increases upon *β*-catenin knockdown. These findings provide novel insight into the *β*-catenin/TCF-dependent mechanisms of CRC carcinogenesis, suggesting that the relative amounts of *β*-catenin and those of different TCFs may ultimately dictate or relieve a default repression of *nherf1* gene, in keeping with the notion that TCFs function as powerful transcriptional activators or repressors [40]. NHERF1 expression is known to be negatively regulated by histone deacetylases [41], and was correlated with increasing levels of HIF1*α* (hypoxia-inducible factor 1*α*) [27, 31] that competes with TCF4 for direct binding to *β*-catenin under hypoxia [42]. Thus, it seems conceivable that either epigenetic changes and/or microenvironment cues may further integrate different molecular factors, within or outside the central Wnt/*β*-catenin cascade, to modulate NHERF1 expression, as well as the phenotypic heterogeneity observed in primary human CRC [8, 11].

To our knowledge, this is the first report disclosing NHERF1 as a major survival driver in settings of *β*-catenin-depleted CRC cells which adopt a cytostatic viable phenotype and a unique proteomic signature centred around autophagy and energy metabolism processes (i.e., ribosome biogenesis, spliceosome and RNA transport, oxidative stress and fatty acid degradation). Accordingly, recent evidence earmark NHERF1 as an autophagy regulator in breast cancers via its influence on the ubiquitin-dependent degradation of BECN1, a critical component of the autophagic core lipid kinase complex [43]. Moreover, NHERF1 was recently implicated in the oxidative stress response regulation in liver cancer cells via the recruitment of the MAPK-activated protein kinase 2 (MK2) that belongs to the stress-induced p38 MAPK pathway [44].

In our hands, genetic or pharmacological targeting of NHERF1 was sufficient to prime an ‘autophagy-to-apoptosis switch’ that strikingly impacted the fate of CRC cells during *β*-catenin knockdown. These data were remarkably consistent across two human CRC cell lines harboring different Wnt/*β*-catenin pathway mutations in *APC/KRAS* or *β-*catenin*/KRAS* genes, thus excluding any clonal effects. Mechanistically, combined targeting of NHERF1 and *β*-catenin triggered the activation of Caspase-3 and the cleavage of PARP also abrogating the hyper-activation of ERK1/2, the accumulation of autophagy-related proteins like Beclin-1 (BECN1), Rab7 or its effector RILP elicited by single *β*-catenin knockdown. These molecular changes may relatively explain why a double *β*-catenin/NHERF1-inhibitory strategy may be a fruitful mechanism-based strategy to augment apoptotic death of CRC cells refractory to Wnt/*β*-catenin-targeted therapeutics [3, 5, 6].

However, as physiological or pathological roles of NHERF1 strictly depend on its subcellular localization, targeted approaches aiming at modulating NHERF1 activity, rather than its overall expression, would be preferred to preserve the normal functions of this versatile protein. By far, particular attention has been paid to the NHERF1/PDZ1-domain that governs its membrane recruitment/displacement through a transient phosphorylation switch [7, 21–23]. By using a rational virtual screening, we herein validated RS5517 as a novel NHERF1/PDZ1 ligand that abrogates its nuclear entry exhibiting a remarkable cytotoxicity on *β*-catenin-depleted CRC cells which highly re-express NHERF1.

Although, further studies will exploit the safety profile of RS5517 in vivo, understanding that *β*-catenin signaling negatively regulates NHERF1 in CRC tumorigenesis will trigger the refinement of Wnt-targeted approaches to overcome the therapeutic resistance occurring at early disease stages.

## Materials and methods

### Cells cultures and reagents

Colorectal cancer (CRC) cell lines Ls174T and DLD-1 were purchased from the American Type Culture Collection (Manassas, VA, USA) and routinely assessed to check their identity [45]. Cells were stably transfected with Doxycycline hyclate (Dox)-inducible short hairpin RNA (shRNA) for *β*-catenin (Ls174Tsh*β*-Cat and DLD1sh*β*-Cat) or a scramble control shRNA (shCtr), as previously described [3, 45]. To obtain NHERF1 downregulation, Ls174Tsh*β*-Cat and DLD1sh*β*-Cat were transfected with 250 nM of NHERF1 shRNA (shNHERF1) or a scramble control shRNA (shCtr) (sc-156113) (Santa Cruz Biotechnology, CA, USA), according to the manufacturer’s instructions.

FH535 (Calbiochem, San Diego, CA) and pyrvinium pamoate (Sigma-Aldrich) were resuspended in dimethyl-sulfoxide (DMSO, Sigma-Aldrich).

### RT-PCR

Total RNA of Ls174Tsh*β*-Cat and DLD1sh*β*-Cat cells was isolated using Trizol reagent (Invitrogen, Carlsbad, CA). The *Nherf1* mRNA levels were determined using an RT-PCR kit (New England Biolabs, Beverly, MA) and the following primers: forward 5′-CCCAGTGGCTATGGCTTCAA-3′ and reverse 5′-GAAGTCTAGGATGGGGTCGG-3′. The primers for β-actin were: forward 5′-CCACGGCTGCTTCCAGCTCC-3′ and reverse 5′-GGAGGGCCGACTCGTCAT-3′. The relative *Nherf1* mRNA abundance versus β-actin mRNA was quantified by Image J analysis.

### Chromatin immunoprecipitation (ChIP) assay

A CHIP-KIP, including an anti-TCF4 antibody, a mouse IgG control and *Gapdh* promoter primers was from Millipore (#17-10109). An anti-TCF1 antibody (clone 7H3) was also from Millipore. TCF-associated DNA immunoprecipitates were verified by qPCR using SYBR Green Mix (TaKaRa) and *Nherf1* promoter primers as follows: *Nherf1* 5′-CCTCCGTCTTAATTCTCGAG-3′ (forward) and 5′-CCTTCACCTTCACAAACAAT-3′ (reverse). Data are reported as percent input of each IP sample relative to input chromatin for each amplicon and ChIP sample.

### Immunofluorescence staining

Cells were assayed using an anti-NHERF1 (1:500; ThermoFisher, Rockford, IL) or *β*-catenin primary antibody (1:500; BD Transduction Laboratories, Lexington, KY) followed by Alexa Fluor 488-conjugated goat anti-rabbit IgG-FITC or a goat anti-rabbit IgG-TRITC (1:1000; Molecular Probes, Inc., Eugene, OR) secondary antibodies, and then analyzed using a confocal laser scanning microscope (LSM 710, Zeiss, Germany), equipped with a 60 oil-immersion objective and a spatial resolution of 200 nm in *x*–*y* and 100 nm in *z*.

### Staining of monodansylcadaverine (MDC)-labeled vacuoles

Cells were stained with 0.05 mM MDC in PBS at 37 °C for 10 min, washed and assayed by fluorescence microscope (Nikon Eclipse TE300, Japan). Images were captured using Image Pro-plus software.

### Cell proliferation and apoptosis assays

Cells (10^5^–50^5^ cells/well) were treated with serial dilutions of RS5517 or DMSO in the absence (−Dox) or presence (+Dox) of Dox in a volume of 200 μl. Cell growth was quantified by MTT (3-[4,5-dimethylthiazol-2-yl]-2,5-diphenyl Tetrazolium bromide) (Sigma-Aldrich) according to manufacturer’s recommendations. For apoptosis assays, the cells were stained with Annexin V-PE (BD-PharMingen, San Diego, CA) or Caspase-Glo assay kit (Promega) and analyzed by flow cytometry.

### Luciferase reporter assays, flow cytometry, and soft agar clonogenic analysis

Cells were analyzed as previously described [45].

### Subcellular fractionation assays

Cells were collected in the subcellular fractionation buffer (SF buffer): 250 mM sucrose, 20 mM HEPES (pH 7.4), 10 mM KCl, 1.5 mM MgCl_2_, 1 mM EDTA and EGTA, to which 1 mM DTT and protease/phosphatase inhibitors (1 mM PMSF; 5 mM NaF and 1 mM Na_3_VO_4_) were added at time of use. Lysates were then put on a tube roller (30–50 r.p.m.) at 4 °C for 30 min and centrifuged at 720×*g* at 4 °C for 5 min. Pellet was washed with 500 μl of SF buffer, centrifuged at 720×*g* at 4 °C for 10 min, and dissolved for 15 min in nuclear lysis buffer (NL buffer): 50 mM Tris-HCl (pH 8), 150 Mm NaCl, 1% NP-40, 0.5% sodium deoxycholate, 0.1% SDS, to which 10% glycerol and protease/phosphatase inhibitors were added at time of use. To obtain cytosolic fraction, the supernatant was centrifuged at 10,000×*g* at 4 °C for 10 min and ultracentrifuged at 100,000×*g* at 4 °C for 1 h. To obtain the membrane fraction, the ultracentrifuged pellet was washed with SF buffer and ultracentrifuged at 100,000×*g* at 4 °C for 1 h. Last pellet was dissolved in NL buffer and sonicated on ice.

### Pulse-chase analysis, immunoprecipitation, and western blotting

Cells were assayed as previously described [45]. Primary antibodies were as follows: total *β*-catenin was from BD Transduction Laboratories, total NHERF1 from ThermoFisher, total c-Myc, cyclin D1, p27, Rab7, RILP, V1G1, Cathepsin D, and *α*-Tubulin were from Santa Cruz Biotechnology Inc. (Santa Cruz, CA), total and phosphorylated pERK1/2 (pThr202/Tyr204) MAPK, total AKT, LC3, Beclin-1 (BECN1), and PARP were from Millipore.

### Sample preparation and mass spectrometry evaluation

Quantity of 20 μg of whole-protein extracts prepared using Illustra TriplePrep kit (GE Healthcare) were diluted 10-fold in 8 M urea in 0.1 M Tris-HCl, pH 8.5, filtered into the Microcon Ultracel YM-30 devices (Millipore), and centrifuged at 14,000×*g* for 15 min. Samples were then further diluted in 8 M urea, centrifuged again, reduced in 10 mM DTT for 30 min, and then alkylated in 50 mM IAM for 20 min. After four washes (2 in 8 M urea and 2 in 50 mM NH_4_HCO_3_), trypsin solution was added in an enzyme-to-protein ratio of 1:100 w/w, and samples were maintained at 37 °C for 16 h. Peptides were centrifuged and acidified by trifluoroacetic acid, desalted-concentrated on C-18 ZipTip (Millipore), dried under vacuum and then resuspended in 20 µl of ACN/H_2_O (FA 0.1%) (2:98, v/v). Separation was obtained using an EASY-nLC 1000 UPLC (Thermo Scientific) through 75 mm × 2 cm pre-column with nanoViper fittings (Acclaim pepMap 100, C18, 2 µm, Thermo Scientific) and 50 mm ID × 150 mm analytical column with nanoViper fittings (Acclaim PepMap RSLC, C18, 2 µm, Thermo Scientific). Elution was carried out over 120 min by using a 2-h gradient of ACN. The Q-Exactive instrument (Thermo Scientific) was set up to a spray voltage of 1.6 kV and the survey scans were taken at 70,000 FWHM (at m/z 400) resolving power in positive ion mode with a scan range from 300 to 1600 m/z.

### Database searching and bioinformatics analysis

Q-Exactive spectra were processed using the MaxQuant proteomics software (version 1.5.3.8) and matched using the Andromeda algorithm [46]. Trypsin was used as enzyme with two missed cleavages allowed. N-terminal acetylation and methionine oxidation were variable modifications, while carbamidomethylation of cysteines was a fixed modification. An initial mass spectra accuracy of 6 p.p.m. was selected, and the MS/MS tolerance was 20 p.p.m. for the HCD data with a false discovery rate of 1% for peptides and proteins identification. The MaxLFQ algorithm was used for assessing relative, label-free quantification of the proteins [47].

### Chemical synthesis of the RS5517 derivative

RS5517, namely 3-benzyl-5-chloro-N-(4-(hydroxymethyl)phenyl)-1H-indole-2-carboxamide, was synthesized by coupling reaction of 3-benzyl-5-chloro-1H-indole-2-carboxylic acid with protected (4-(hydroxymethyl)aniline in the presence of PyBOP reagent and triethylamine in DMF. The product was deprotected by reaction with tetrabutylammonium fluoride solution (1 M in anhydrous THF).

### Virtual screening studies

The NHERF1 PDZ1 domain was retrieved from the Protein Data Bank, under the PDB accession code 21GQ4 and the substrates (DSLL and ETVM) were drawn by Maestro (Schrödinger Release 2016-4: Maestro, Schrödinger, LLC, New York, NY, 2016) and analysed by molecular dynamics with Amber 12 [48]. The minimized structure was solvated in a periodic octahedron simulation box using TIP3P water molecules, providing a minimum of 10 Å of water between the protein surface and any periodic box edge. Following minimization, the entire system was heated to 298 K (20 ps), and the simulation was conducted at 298 K with constant pressure, periodic boundary condition and shake bond length condition (ntc = 2). Trajectories analysis were carried out by the *cpptraj* modules [25]. This led to 5 structures for each complex which were used to draw a consensus pharmacophore model by Phase [26]. An in-house library of about 6000 compounds were docked into the binding site of each structures by Plants [49]. Structures fitting the pharmacophore queries were selected for biological evaluation. Picture was depicted by Pymol (PyMOL version 1.2r1. DeLanoScientificLLC:SanCarlos, CA).

### In vitro binding studies

A NHERF1 PDZ1 subcloned in pETG41 plasmid was kindly obtained from Dr. Nicolas Wolff (Pasteur Institute, Paris, France). A site-directed mutagenesis by Quickchange Lighting mutagenesis kit was confirmed by DNA sequencing. A dansylated peptide relative to the C-terminal portion of the β2-adrenergic receptor, *D*-NDSLL, was from JPT Peptide Technologies (Berlin, Germany) and purified using HPLC. Equilibrium binding experiments were carried out in the presence of 50 mM Na phosphate buffer, pH 7.2, 300 mM NaCl at 25 °C by monitoring fluorescence upon exciting the sample at 280 nm and measuring emission on a Fluoromax single-photon spectrofluorometer (Jobin-Yvon, NJ, USA) [50].

### Statistical analysis

Data are reported as the mean ± SEM. Significance levels were determined with the Student's *t*-test. Proteomic statistical analysis was perfomed with the Perseus software (version 1.5.2.4). Multiple-samples tests were performed using ANOVA test by using a false discovery rate threshold of 0.05 and preserving grouping in randomization. Hierarchical clustering of proteins was performed in Perseus on logarithmized intensities after *z*-score normalization of the data, using Euclidean distances. Gene Ontology (GO) and Kyoto Encyclopedia of Genes and Genomes (KEGG) analysis of our protein dataset was performed by STRING version 10 (http://string-db.org) (Nucleic Acids Res 2015; 43(Database issue):D447-52. doi: 10.1093/nar/gku1003).

## Electronic supplementary material

Supplementary Figure S1, S2, S3(PDF 6310 kb)
