# Supplementary material for: β-catenin knockdown promotes NHERF1-mediated survival of colorectal cancer cells: implications for a double-targeted therapy
Source: Oncogene. 2018 Mar 19;37(24):3301–16. doi: 10.1038/s41388-018-0170-y (PMC6002344; doi:10.1038/s41388-018-0170-y)
Supplement: Supplementary file 1 — Supplementary Figure S1, S2, S3(PDF 6310 kb) [file 41388_2018_170_MOESM1_ESM.pdf]

Supplementary Figures

β-Catenin knockdown promotes NHERF1-mediated survival of colorectal cancer cells: implications for a double-targeted therapy

<u>Running title</u>: **Oncogenic β-Catenin signaling negatively regulates NHERF1**

<u>Keywords</u>: Wnt/β-Catenin signaling, colon cancer, NHERF1, PDZ domain, target therapy

Concetta Saponaro[1,2], Sara Sergio[1,3], Antonio Coluccia[4], Maria De Luca[3], Giuseppe La Regina[4], Luca Mologni[5], Valeria Famiglini[4], Valentina Naccarato[4], Daniela Bonetti[4], Candice Gautier[4], Stefano Gianni[6], Daniele Vergara[1,3], Michel Salzet[7], Isabelle Fournier[7], Cecilia Bucci[3], Romano Silvestri[4], Carlo Gambacorti Passerini[5], Michele Maffia[1,3], Addolorata Maria Luce Coluccia[1,3]

[1] *Laboratory of Clinical Proteomics, Giovanni Paolo II Oncology Hospital, I-73100 Lecce, Italy*
[2] *Functional Biomorphology Laboratory, IRCCS Istituto Tumori "Giovanni Paolo II", Bari, Italy*
[3] *Department of Biological and Environmental Sciences and Technologies, University of Salento, I-73100 Lecce, Italy*
[4] *Department of Drug Chemistry and Technologies, Sapienza University of Rome, Laboratory affiliated to Istituto Pasteur Italia – Fondazione Cenci Bolognetti, Piazzale Aldo Moro 5, I-00185 Roma, Italy*
[5] *Department of Clinical Medicine, San Gerardo Hospital, University of Milano-Bicocca, I-*20052 *Monza, Italy*
[6] *Department of Biochemistry, Sapienza University of Rome, Laboratory affiliated to Istituto Pasteur Italia – Fondazione Cenci Bolognetti, Piazzale Aldo Moro 5, I-00185 Roma, Italy*
[7] *U1192-Laboratoire Protéomique, Réponse Inflammatoire et Spectrométrie de Masse (PRISM), F-59000 Lille, France*

<u>Figures</u>: **9** / <u>Table</u>: **1** / <u>Supplementary Figures</u>: **3** / <u>References</u>: **50**

The authors declare no competing financial conflicts of interest.

Corresponding author: Addolorata Maria Luce Coluccia, Ph.D.

Laboratory of Clinical Proteomics 'Giovanni Paolo II' Oncology Hospital
University of Salento - Piazza Muratore, 1, I-73100 Lecce, Italy
Tel.: +39 0832661915; e-mail: malu.coluccia@unisalento.it

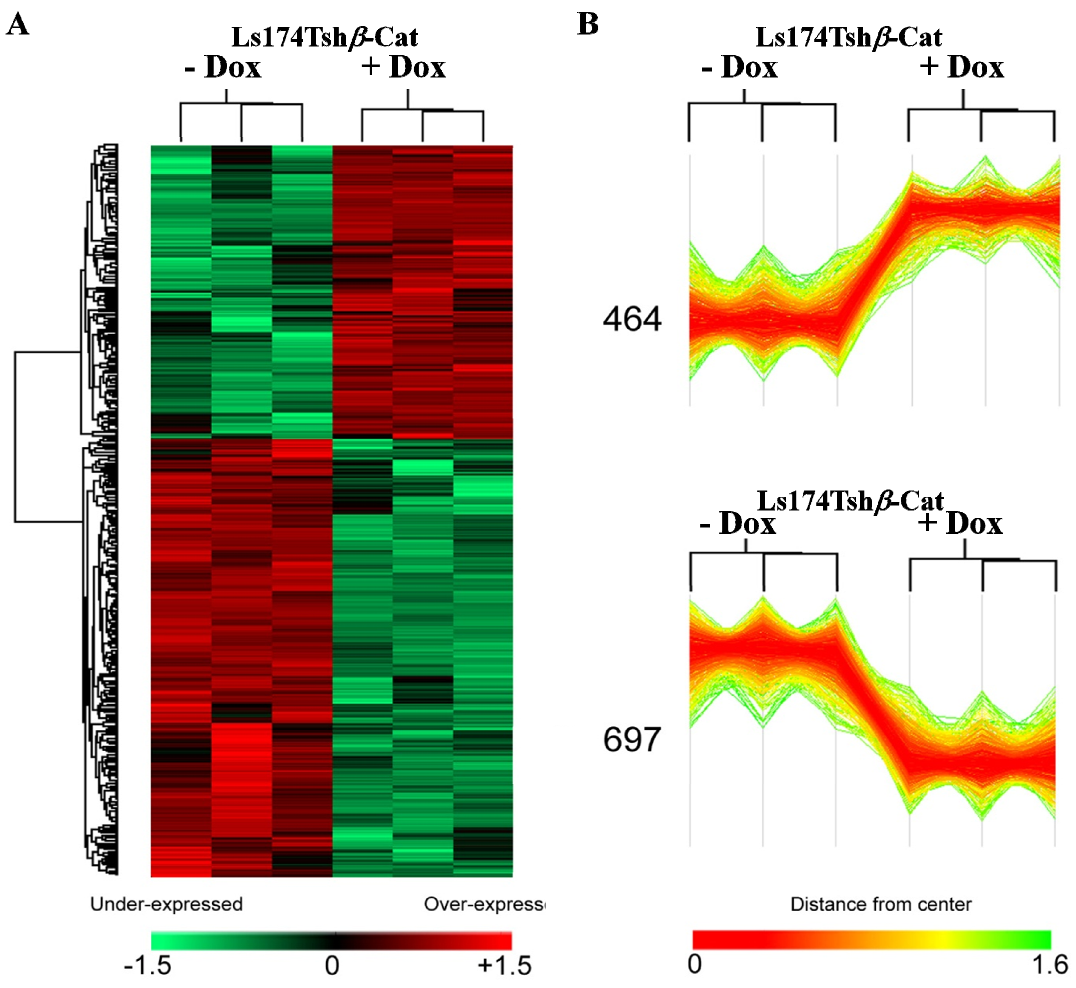

Saponaro C. et al.; **Supplementary Figure S1**

Supplementary Figure S1. LC-MS/MS analysis of *β*-Catenin depleted CRC cells. (A) Ls174Tsh*β*-Cat cells were cultured in the absence or presence of Dox (-Dox; n = 3/+Dox; n = 3) for 5 days and analysed for the identification of differentially expressed proteins. Heat map of proteins with different regulation profiles as determined after label free quantification highlighting the presence of 2 main protein clusters. Hierarchical clustering of proteins was performed in Perseus on logarithmized intensities after *z*-score normalization of the data, using Euclidean distances. In each cluster, proteins down-regulated are presented in green, and proteins up-regulated are shown in red. (B) Two main clusters extracted from A. The windows contain the expression profiles of the

proteins within clusters. The number of differentially expressed proteins in each cluster is also depicted.

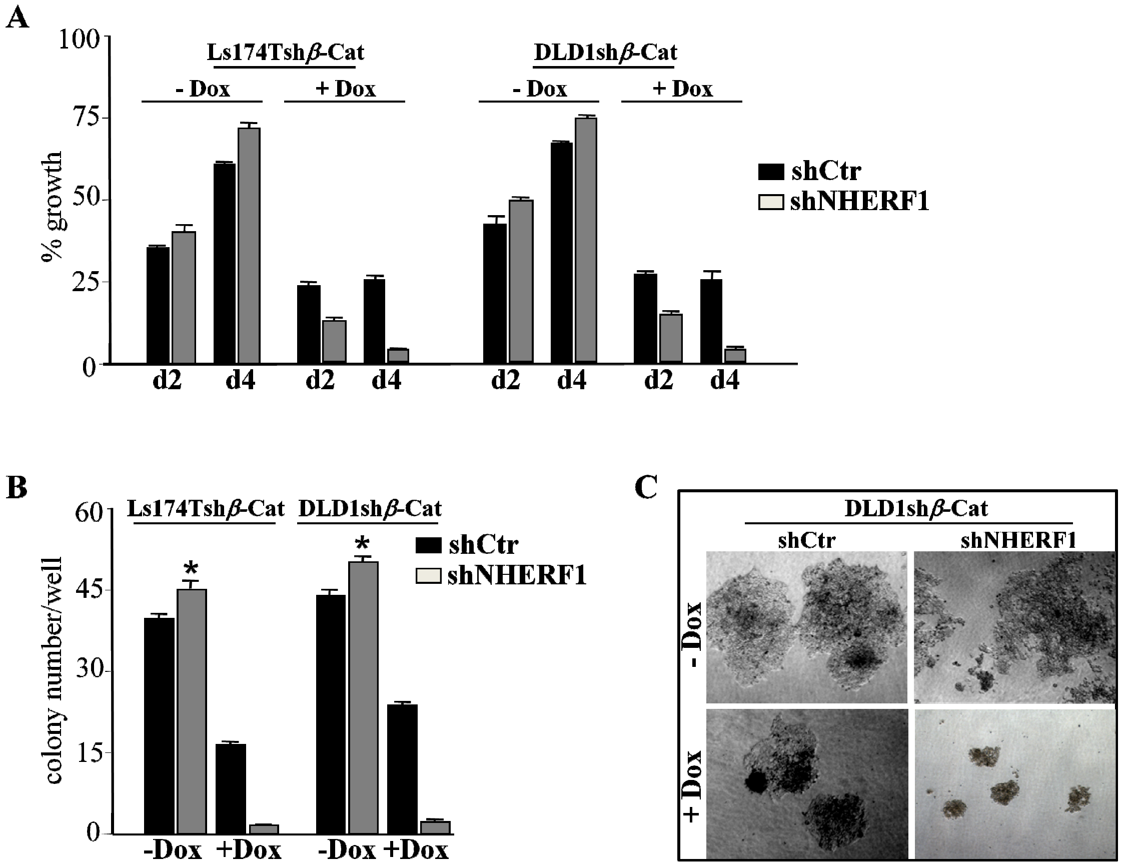

Saponaro C. et al.; **Supplementary Figure S2**

Supplementary Figure S2. NHERF1 shRNA-knockdown confers a moderate growth advantage to CRC cells and promotes a sprouted/invasive phenotype. Subconfluent Ls174T or DLD1 cells stably harbouring a doxycycline (Dox)-inducible shRNA for *β*-Catenin (Ls174Tsh*β*-Cat or DLD1sh*β*-Cat) were further transiently transfected with 250nM of NHERF1 targeted shRNAs (shNHERF1) or scramble shRNAs as control (shCTR). (A) Proliferation assays of Ls174Tsh*β*-Cat or DLD1sh*β*-Cat transfected with the indicated shRNA and cultured in the absence or presence of Dox (-Dox/+Dox) for 2 and 4 days. Data were expressed as % of growth inhibition. (B) Number of

colonies formed in soft agar by Ls174Tshβ-Cat or DLD1shβ-Cat transfected with the indicated shRNA and cultured in the absence or presence of Dox (-Dox/+Dox) for 14 days. The values are presented as mean ± SD of three independent experiments (*=P<0.05). (C) A representative cell colony image was captured for the indicted treatment condition by using a camera attached to an inverted Olympus IX51 microscope.

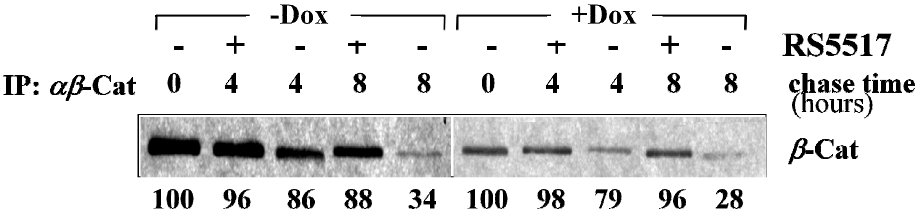

Saponaro C. et al.; **Supplementary Figure S3**

Supplementary Figure S3. RS5517 delays the protein degradation rate of β-Catenin. Subconfluent DLD1Tshβ-Cat cells treated with 10μM RS5517 in the absence or concomitant presence of 2μg/mL of Dox (-Dox/+Dox) for 3 days were then labeled with [^{35}S]-methionine and then chased with complete nonradioactive medium for additional 4 or 8 hours. Cells were then lysed and immunoprecipitated for β-Catenin. The results were analyzed by densitometry and expressed as a percentage of the intensity value at time 0 for cells cultured without or with Dox (-Dox/+Dox), respectively.
